# HIV self-testing services for female sex workers, Malawi and Zimbabwe 

**DOI:** 10.2471/BLT.18.223560

**Published:** 2019-09-03

**Authors:** Sue Napierala, Nicola Ann Desmond, Moses K Kumwenda, Mary Tumushime, Euphemia L Sibanda, Pitchaya Indravudh, Karin Hatzold, Cheryl Case Johnson, Rachel C Baggaley, Liz Corbett, Frances M Cowan

**Affiliations:** a Women’s Global Health Imperative, RTI International, 351 California Street, Suite 500 San Francisco, CA 94104, United States of America.; b Department of International Public Health, Liverpool School of Tropical Medicine, Liverpool, England.; c Malawi-Liverpool-Wellcome Trust Clinical Research Programme, Blantyre, Malawi.; d Centre for Sexual Health and HIV/AIDS Research, Harare, Zimbabwe.; e Population Services International, Harare, Zimbabwe.; f Department of HIV, World Health Organization, Geneva, Switzerland.; g Department of Clinical Research, London School of Hygiene and Tropical Medicine, London, England.

## Abstract

**Objective:**

To present findings from implementation and scale-up of human immunodeficiency virus (HIV) self-testing programmes for female sex workers in Malawi and Zimbabwe, 2013–2018.

**Methods:**

In Zimbabwe, we carried out formative research to assess the acceptability and accuracy of HIV self-testing. During implementation we evaluated sex workers’ preferences for, and feasibility of, distribution of test kits before the programme was scaled-up. In Malawi, we conducted a rapid ethnographic assessment to explore the context and needs of female sex workers and resources available, leading to a workshop to define the distribution approach for test kits. Once distribution was implemented, we conducted a process evaluation and established a system for monitoring social harm.

**Findings:**

In Zimbabwe, female sex workers were able to accurately self-test. The preference study helped to refine systems for national scale-up through existing services for female sex workers. The qualitative data helped to identify additional distribution strategies and mediate potential social harm to women. In Malawi, peer distribution of test kits was the preferred strategy. We identified some incidents of social harm among peer distributors and female sex workers, as well as supply-side barriers to implementation which hindered uptake of testing.

**Conclusion:**

Involving female sex workers in planning and ongoing implementation of HIV self-testing is essential, along with strategies to mitigate potential harm. Optimal strategies for distribution and post-test support are context-specific and need to consider existing support for female sex workers and levels of trust and cohesion within their communities.

## Résumé

**Objectif:**

Présenter les résultats de la mise en œuvre et de l’élargissement de programmes d'autodépistage du virus de l'immunodéficience humaine (VIH) pour les travailleuses du sexe au Malawi et au Zimbabwe entre 2013 et 2018.

**Méthodes:**

Au Zimbabwe, nous avons réalisé une recherche préparatoire pour évaluer l'acceptabilité et la précision de l'autotest du VIH. Au cours de la mise en œuvre, nous avons évalué la faisabilité et les préférences des travailleuses du sexe à l’égard de la distribution des trousses de dépistage avant d'élargir le programme. Au Malawi, nous avons réalisé une évaluation ethnographique rapide pour étudier le contexte, les besoins des travailleuses du sexe et les ressources disponibles, ce qui a donné lieu à un atelier dont l'objectif était de définir la méthode de distribution des trousses de dépistage. Une fois la distribution mise en place, nous avons évalué le processus et établi un système de suivi des dommages sociaux.

**Résultats:**

Au Zimbabwe, les travailleuses du sexe étaient en mesure de procéder à un autodépistage précis. L'étude des préférences a permis de perfectionner les systèmes en vue d'un développement national par l'intermédiaire des services existants pour les travailleuses du sexe. Les données qualitatives ont aidé à définir d'autres stratégies de distribution et les dommages sociaux indirects que peuvent subir les femmes. Au Malawi, la distribution de trousses de dépistage par des pairs a été la stratégie privilégiée. Nous avons identifié quelques cas de dommages sociaux entre des travailleuses du sexe assurant la distribution et d'autres travailleuses du sexe, ainsi que des obstacles du côté de l'offre qui ont limité l'utilisation du test.

**Conclusion:**

La participation des travailleuses du sexe à la planification et à la mise en œuvre continue de l'autodépistage du VIH est essentielle, de même que les stratégies visant à réduire les dommages potentiels. Pour être optimales, les stratégies en matière de distribution et de soutien post-test doivent tenir compte du contexte, du soutien existant apporté aux travailleuses du sexe et du degré de confiance et de cohésion au sein de leurs communautés.

## Resumen

**Objetivo:**

Presentar los resultados de la implementación y ampliación de los programas de autodiagnóstico del virus de la inmunodeficiencia humana (VIH) para trabajadoras sexuales en Malawi y Zimbabwe, 2013-2018.

**Métodos:**

En Zimbabwe, se llevó a cabo una investigación formativa para evaluar la aceptabilidad y exactitud del autodiagnóstico del VIH. Durante la implementación, se evaluaron las preferencias de las trabajadoras sexuales y la viabilidad de la distribución de los kits de pruebas antes de ampliar el programa. En Malawi, se realizó una evaluación etnográfica rápida para explorar el contexto y las necesidades de las trabajadoras sexuales y los recursos disponibles, que condujo a un taller para definir el enfoque de distribución de los kits de pruebas. Una vez implementada la distribución, se llevó a cabo una evaluación del proceso y se estableció un sistema para el seguimiento de los daños sociales.

**Resultados:**

En Zimbabwe, las trabajadoras sexuales pudieron autoevaluarse con precisión. El estudio de preferencias contribuyó a perfeccionar los sistemas de ampliación nacional mediante los servicios existentes para las trabajadoras sexuales. Los datos cualitativos ayudaron a identificar estrategias de distribución adicionales y a mediar en posibles daños sociales para las mujeres. En Malawi, la estrategia preferida fue la distribución de kits de pruebas entre pares. Se identificaron algunos incidentes de daño social entre distribuidores pares y trabajadoras sexuales, así como barreras de suministro para la implementación que dificultaban la realización de las pruebas.

**Conclusión:**

Es esencial involucrar a las trabajadoras sexuales en la planificación y la implementación continua del autodiagnóstico del VIH, junto con estrategias para reducir los daños potenciales. Las estrategias óptimas para la distribución y el apoyo posterior a las pruebas son específicas para cada contexto y deben tener en cuenta el apoyo existente a las trabajadoras sexuales y los niveles de confianza y cohesión dentro de sus comunidades.

## ملخص

**الغرض:**

طرح النتائج المترتبة على تنفيذ برامج الفحص الذاتي لفيروس نقص المناعة البشرية (HIV) وتوسيع نطاقها، وذلك على العاملات في المجال الجنسي في ملاوي وزمبابوي، خلال الفترة من 2013 إلى 2018.

**الطريقة:**

قمنا في زيمبابوي بإجراء بحثاً مركباً لتقييم إمكانية قبول الفحص الذاتي لفيروس نقص المناعة البشرية، وكذلك مدى دقة هذا الفحص. وقمنا أثناء التنفيذ بتقييم تفضيلات العاملات في المجال الجنسي تجاه توزيع أطقم الفحص الذاتي، ومدى جدوى ذلك، قبل أن يتم توسيع البرنامج. أما في ملاوي، فقد قمنا بإجراء تقييم إثنوغرافي سريع لاستكشاف سياق واحتياجات العاملات في المجال الجنسي والموارد المتاحة، مما أدى إلى ورشة عمل لتحديد أسلوب توزيع أطقم الفحص. وبمجرد إتمام التوزيع، قمنا بإجراء تقييم للعملية وأنشأنا نظامًا لمراقبة التضرر الاجتماعي.

**النتائج:**

تمكن العاملات في المجال الجنسي في زمبابوي من إجراء الفحص الذاتي بدقة. ساعدت دراسة التفضيلات في تحسين الأنظمة الخاصة بالتوسع الوطني من خلال الخدمات الحالية للعاملات في المجال الجنسي. ساعدت البيانات النوعية في تحديد استراتيجيات التوزيع الإضافية، والتوسط في التضرر الاجتماعي المحتمل للسيدات. وفي ملاوي، كان توزيع الأقران لأطقم الفحص هو الاستراتيجية المفضلة. قمنا بتحديد بعض حوادث التضرر الاجتماعي بين الموزعين من الأقران والعاملات في المجال الجنسي، وكذلك الحواجز من جانب الإمداد في سبيل التنفيذ، والتي أعاقت انتشار الفحص.

**الاستنتاج:**

إن إشراك العاملات في المجال الجنسي في التخطيط والتنفيذ المستمر للفحص الذاتي لفيروس نقص المناعة البشرية، لهو أمر ضروري، وذلك إلى جانب استراتيجيات تخفيف الضرر المحتمل. تعتمد الاستراتيجيات المثلى للتوزيع، ودعم ما بعد الفحص، على السياق، وهي بحاجة إلى النظر في الدعم الحالي للعاملات في المجال الجنسي، ومستويات.

## 摘要

**目的:**

旨在介绍 2013 年至 2018 年马拉维和津巴布韦针对女性性工作者实施与推广人体免疫缺损病毒（艾滋病毒）自我检测方案的调查结果。

**方法:**

我们在津巴布韦境内开展了形成性研究，以评估艾滋病毒自我检测的可接受性和准确性。推广方案之前，我们在实施过程中评估了性工作者对检测试剂盒分发的偏好与可行性。我们在马拉维进行了一次快速的人种学评估，以探讨女性性工作者的背景与需求以及可用的资源，并为此举办了一场研讨会以确定检测试剂盒的分发方法。实施分发后，我们进行了流程评估并建立了一个社会危害监测系统。

**结果:**

在津巴布韦境内，女性性工作者能够准确地进行自我检测。这项偏好研究利用为女性性工作者提供的现有服务，帮助完善该系统在全国范围内推广。对于女性而言，定性数据有助于制定其他分发策略并调解潜在的社会危害。在马拉维境内，同行之间分发检测试剂盒是首选的策略。我们在同行分发者与女性性工作者之间发现了一些社会危害事件，以及供应方面的实施障碍，这妨碍了检测试剂盒的分发。

**结论:**

让女性性工作者参与规划和持续实施艾滋病毒自我检测至关重要，同时还要制定减轻潜在危害的策略。最佳的分发与检测后支持策略取决于具体情况并且需要考虑对女性性工作者的现有支持及其社区内的信任和凝聚力程度。****

## Резюме

**Цель:**

Представить результаты внедрения и расширения сферы действия программ по самостоятельному тестированию на наличие вируса иммунодефицита человека (ВИЧ) среди работниц секс-индустрии в Малави и Зимбабве в период с 2013 по 2018 год.

**Методы:**

В Зимбабве авторы провели формативное исследование для оценки приемлемости и точности результатов самостоятельного тестирования на ВИЧ. Во время осуществления программы (до начала расширения сферы ее действия) оценивались предпочтения работниц секс-индустрии относительно распространения наборов для самотестирования и возможности осуществления их пожеланий. В Малави авторы провели ускоренную этнографическую оценку для изучения контекста и потребностей работниц секс-индустрии, а также для оценки имеющихся ресурсов. В результате был проведен семинар для определения подхода к распространению наборов для самотестирования. Как только наборы были распространены, авторы провели оценку процесса и разработали систему мониторинга социального вреда.

**Результаты:**

Работницы секс-индустрии в Зимбабве были в состоянии точно выполнить самостоятельное тестирование. Исследование предпочтений помогло уточнить систему расширения сферы действия программы в национальном масштабе, которая использовала уже имеющиеся возможности для работниц секс-индустрии. Данные качественного характера помогли выявить дополнительные стратегии распространения наборов для самотестирования и уменьшить потенциальный социальный вред для женщин. В Малави предпочтительной стратегией распространения наборов для самотестирования было распределение через самих работниц. Авторы выявили некоторые случаи социального вреда, имевшие место среди распространителей наборов для самотестирования и работниц секс-индустрии. Имелись также препятствия со стороны поставщиков, которые снижали эффективность работы программы.

**Вывод:**

Вовлечение работниц секс-индустрии в процесс планирования и постоянного осуществления самотестирования на ВИЧ имеет чрезвычайную важность наряду с разработкой стратегий уменьшения потенциального социального вреда. Оптимальные стратегии распространения тестов и последующей поддержки зависят от контекста. Необходимо учитывать наличие имеющейся поддержки работниц секс-индустрии, а также уровень доверия и сплоченности в их сообществах.****

## Introduction

Female sex workers are a marginalized group who are disproportionately affected by human immunodeficiency virus (HIV) infection.^1^ Despite reduction in HIV infections in the general population, the prevalence and incidence of HIV among female sex workers remain extremely high.^2^^–^^4^ In sub-Saharan Africa the proportion of HIV infection in the general female population that is attributable to sex work is estimated to be 17.8% (upper to lower estimates obtained from sensitivity analysis: 13.6–22.1%).^5^^,^^6^ Due to multiple barriers, female sex workers underuse HIV services, including HIV testing services.^4^^,^^7^^,^^8^ Increasing the engagement of female sex workers in HIV testing services is the first step towards prevention and care services that will minimize health inequity. Increasing timely initiation of antiretroviral therapy (ART) should reduce morbidity for these women and onward HIV transmission to partners and clients of sex workers.

Self-testing for HIV may be particularly appropriate for female sex workers as an additional testing option. Female sex workers need, and often desire, frequent re-testing and typically have high knowledge of HIV. HIV self-testing has been shown to be acceptable and feasible for female sex workers and to increase the uptake of testing. Self-testing may improve the efficiency of testing among both female sex workers and health-care providers.^9^^,^^10^ Modelling studies in Zimbabwe demonstrated that increasing HIV testing among female sex workers could be one of the most cost–effective uses of HIV self-testing.^11^ Secondary distribution of kits to other female sex workers and sexual partners is also feasible.^12^^,^^13^ A study in Uganda showed that, similar to general populations, HIV test sensitivity among female sex workers can be low when test kits are distributed without support for carrying out the self-test.^9^ A concern for this high HIV-incidence population is the limited analytical sensitivity of HIV self-testing for detecting early infection in female sex workers, especially among those taking pre-exposure prophylaxis.^2^ HIV self-testing may facilitate testing among sex workers’ peer and sexual networks, but this may also make the method more prone to coercive misuse than other testing strategies.

The feasibility of scaling-up HIV self-testing programmes for female sex workers is of interest to national HIV programmes. However, the optimal models for distributing kits and providing support for women needing confirmatory testing remain undefined, and balancing the potential risks and benefits of self-testing is needed in this context. 

We have conducted HIV self-testing implementation research among female sex workers in Malawi and Zimbabwe. Zimbabwe has one of the few nationally scaled programmes for female sex workers in Africa (Sisters with a Voice), established in 2009.^14^ The Sisters clinics are co-located within government services in convenient, discreet locations, with staff trained to maintain a friendly and non-judgemental environment. Services are supported by a network of peer educators. To date over 65 000 women have received services nationwide. The National HIV/AIDS Programme in Malawi, by contrast, has focused on meeting general population needs through public clinic-based services that exemplify a public health approach. There has been little focus on the specific needs of key populations in Malawi until dedicated funding for services for female sex workers became available in 2015.

We present and compare findings from programmes for implementation of HIV self-testing for female sex workers in these two countries. We discuss similarities and differences, lessons learnt, knowledge gaps, and the feasibility of scaling-up HIV self-testing programming for female sex workers.

## Methods

The settings for the studies in Malawi and Zimbabwe are shown in Box 1. In Table 1 we summarize the research methods and dates in the development of HIV self-testing programmes by the three phases: (i) formative assessment, (ii) implementation and (iii) scale-up. All research was reviewed and approved by the relevant institutional review boards for each country.

Box 1 **Settings of the studies HIV self-testing services for female sex workers in Malawi and Zimbabwe**

In Malawi, research on HIV self-testing has been carried out in collaboration with Pakachere Institute of Health and Development Communication, the implementing partner for female sex workers’ services in Blantyre and Mangochi districts. Pakachere Institute was providing outreach services to female sex workers, including sexually transmitted infection screening and treatment; family planning; HIV testing services; and HIV prevention services such as distribution of condoms and lubricants. Services were provided through funding from the Malawi Linkages project at three female sex workers’ drop-in clinics operating out of primary care clinics. Outreach was mainly delivered through peer educators.

Research on HIV self-testing in Zimbabwe has been conducted within the Sisters with a Voice programme by several investigators in partnership with Zimbabwe’s Ministry of Health and Child Care. Self-testing is offered through facilities with the option of self-testing on site or taking tests kits away. The programme combines community mobilization with risk-reduction counselling and efforts to increase HIV testing, alongside provision of condoms and other sexual health services. For those testing HIV-positive the programme assists with referral to government ART services and provides support for treatment adherence. For those testing negative, women are encouraged to engage in routine testing and, when available, are offered referral for pre-exposure prophylaxis. The HIV self-testing programme was implemented within this framework.

HIV: human immunodeficiency virus.

**Table 1:** Methods used in studies of HIV self-testing programmes for female sex workers in Malawi and Zimbabwe, 2013–2018

Objective, by phase and country	Study design	Inclusion criteria and recruitment of participants	Outcomes studied and analyses	Time period	Results and conclusions
**I. Formative assessment phase**					
**Zimbabwe**					
Assessment of acceptability of HIV self-testing among female sex workers	Representative survey of female sex workers participating in the Sisters Antiretroviral Therapy Programme for Prevention of HIV, an Integrated Response (SAPPH-Ire) trial.^15^ Baseline survey conducted in 14 communities, with 5 questions about hypothetical acceptability of HIV self-testing	Sample of 2722 female sex workers recruited via respondent-driven sampling. Women had lived and worked in the community for the preceding 6 months; had exchanged sex for money or goods in the past 30 days; were 18 years of age or older	Descriptive analysis of questionnaire data, weighted to take account of respondent-driven sampling recruitment and clustering	Nov 2013–Dec 2013	Study confirmed high acceptability of self-testing; 2069 (76%) of female sex workers were interested in testing themselves for HIV, 1960 (72%) would test more often if HIV self-testing were available. Results also confirmed potential for HIV self-testing programming in this population
Assessment of female sex worker's understanding of instructions for use and accuracy of HIV self-testing	Female sex workers were provided with instructions and video demonstration of HIV self-testing, then conducted self-testing in private. Immediate confirmatory testing was delivered. Confirmatory test was rapid HIV test conducted by health care provider blinded to women’s self-test results, according to the national algorithm. Women were video-recorded while self-testing to review for errors. Structured interviewer-administered questionnaires about HIV self-testing conducted with women pre- and post-test	Convenience sample of 40 female sex workers attending Harare Sisters Clinic; 18 years of age or older	Descriptive analysis of questionnaire data and review of videos. Reported sensitivity and specificity of self-test result compared with confirmatory test result	Aug 2015–Sep 2015	Self-test accuracy was high, with 100% sensitivity and specificity. Results supported feasibility of self-test programming in this population
**Malawi**					
Rapid ethnographic assessment of population sub-types, contexts and needs	Participant observation in purposively selected bars and sex-worker locations. Embedded semi-structured interviews conducted with different groups for formal data collection	Purposive recruitment of 34 female sex workers, 2 peer educators and 101 facility owners	Descriptive reports of triangulated data focusing on context including service hotspots, environments, social interactions and structural conditions for distribution of test kits	Jan–Jun 2016	Study provided contextual data to inform workshop, including sex workers’ behaviours, interactions, preferences and barriers to self-testing
Evaluation of potential HIV self-testing distribution models. Assessment of acceptability of using peer-distributors for HIV self-testing kit distribution	3-day participatory workshop for stakeholders to introduce HIV self-testing, present results of formative research and invite input on the proposed peer-distribution model	Purposive recruitment of stakeholders, including 12 female sex workers, 3 peer educators in peer distribution communities, 5 service providers and one municipal council representative	Evaluation of discussions using an iterative feedback process to determine a consensus on the final HIV self-testing programme model which would reflect local priorities and contexts	Sep 2016	Inclusive, peer distribution model was acceptable to respondents. Results informed refinement of the distribution model and finalization of the proposed intervention
**II. Implementation phase**					
**Zimbabwe**					
Assessment of female sex workers’ preference for HIV self-testing versus standard HIV testing services	Observational study of direct offer of HIV self-testing or standard HIV testing services to all female sex workers of unknown HIV status attending Harare Sisters clinic services. Cohort followed for intended-user intended-setting accuracy.^a^ Interviewer-administered questionnaires conducted with women pre- and post-test	All 607 female sex workers attending the Harare Sisters clinic for HIV testing; mobile phone owners;18 years of age or older; consented to participate	Descriptive analysis of data on participants’ preferences for testing, acceptability of testing and linkage to post-test services. Summary of qualitative results about distribution and support strategies	Nov 2015–Apr 2016	325 female sex workers (54%) opted for HIV self-testing; 313 (96%) self-tested onsite, 97 (99%) of 98 women with a reactive result attended post-test services within 4 weeks. Study informed early implementation of HIV self-testing programming
Qualitative exploration of female sex workers’ views on HIV self-testing	In-depth interviews with female sex workers	Purposive recruitment of a sample of 31 female sex workers from among the 325 who had used self-testing in the observational study	Interviews coded and summarized, using a thematic approach with NVivo software (QSR International, Melbourne, Australia)	Dec 2015–Apr 2016	Study further informed implementation of HIV self-testing programming
Assessment of early implementation of HIV self-testing programme	Routine services included option of HIV self-testing to use onsite or take home	All female sex workers attending one of six Sisters clinics and accepting an offer of HIV testing	Descriptive analysis of participants’ preferences, acceptability of self-testing and linkage to post-test services	Oct 2016–present	Study refined distribution strategies for test kits and assisted start of scale-up
**Malawi**					
Assessment of early implementation of peer distribution model	Peer distributors were trained to provide up to 2 HIV self-testing kits to fellow female sex workers and provided with stock of test kits	28 peer distributors initially recruited and trained from among female sex workers working in three districts: one urban and two rural (12 in Blantyre; 8 in Mulanje; 8 in Chikwawa), including 1 lead peer distributor from each district. Eight distributors (6 in Blantyre; 2 in Mulanje) were dropped due to poor performance and 5 new distributors were trained in their place	Descriptive analysis of participants’ uptake of self-testing and linkage to post-test services	Feb 2017–Jul 2017	Study demonstrated feasibility of peer-distribution model, with 5281 test kits distribution, and highlighted some challenges and limitations
Monitoring and reporting on possible social harms of self-testing	Cohort study with female sex workers reporting experience with HIV self-testing. Conducted over 3 months using pre-test (immediately after HIV self-testing) and post-test (3 months after testing). Study used cross-sectional audio computer-assisted self-interview surveys, 3-month daily event reporting diaries, and serial biographical interviews	265 female sex workers who received HIV self-testing kits through peer-distribution services	Descriptive analysis of social harms, including coercion, forced testing and disclosure, and gender-based violence. Thematic analysis of contextual drivers of social harm	Feb 2017–Nov 2017	Study provided insights on nature of social harm to female sex workers linked to peer distribution of HIV test kits. Harms were low level and primarily concerned coercive testing and forced disclosure of results. No breaches of confidentiality were reported
Process evaluation, focused on HIV self-testing distribution and acceptability	Focus group discussions	Purposive sample of female sex workers who had self-tested (3 focus groups among 21 individuals), peer-distributors taking part in HIV self-testing distribution (1 focus group among 8 individuals)	Thematic content analysis used to code and summarize discussions	Nov 2017–Jan 2018	Female sex workers had positive views on HIV self-testing via peer distribution. Peer-distributors felt respected. Coverage was perceived as restricted
**III. Scale-up phase**					
**Zimbabwe^b^**					
Assessment of scaling-up HIV self-testing programme	Distribution of HIV self-testing kits at 7 clinics and 12 outreach sites. Routine services include distribution of up to 3 additional HIV self-testing kits to anyone testing for HIV to take home for sexual partners	All 19 251 female sex workers attending one of the seven static Sisters clinics or 12 outreach sites and accepting offer of HIV testing	Descriptive analysis of routine programme data, e.g. number of women attending, for what reason, what services were provided	Clinics: Oct 2016–present; outreach: Sep 2017–present; additional kits for secondary distribution: Oct 2017–present	Study found consistent demand for HIV self-testing with 12 071 (63%) female sex workers opting to self-test. There was high preference for onsite self-testing, 11 347 (94%) of female sex workers tested onsite, which facilitates easy linkage to post-test support and care. A minority (8%) of female sex workers took up additional test kits for regular clients and partners
Qualitatively explore possible HIV self-testing service provision beyond Sisters clinics	15 focus group discussions with 7–10 participants each. Focus group discussion were conducted with stakeholders to evaluate additional HIV self-testing distribution strategies. Emphasis was on how to reach those who do not attend Sisters clinics	Purposive selection of participants from 5 stakeholder groups: female sex workers attending Sisters services (6 focus groups with 54 individuals), female sex workers peer educators (6 groups with 55 individuals), female condom sales agents (1 group of 7 individuals), condom-promoting hairdressers (2 groups among 16 individuals) and female sex workers not attending Sisters services (4 groups among 39 individuals)	Discussions coded and summarized using a thematic approach with NVivo software	Sept 2016–Jan 2017; May 2017	Stakeholders rejected some potential distribution strategies for test kits, e.g. distribution by hairdressers and condom promoters. Based on these results next steps will include proceeding with a community-based voucher system targeting hard-to-reach female sex workers and those in their social and sexual networks

HIV: human immunodeficiency virus.

^a^ As per World Health Organization pre-qualification terminology, intended-use intended-setting accuracy study is evaluating implementation under suboptimal, real-life settings.

^b^ Scale-up was done only in Zimbabwe.

### Formative assessment phase

In Zimbabwe, the formative research initially aimed to assess the acceptability and accuracy of HIV self-testing. From November to December 2013 we surveyed a representative sample of female sex workers from 14 sites nationally^16^ and asked about the hypothetical acceptability of HIV self-testing. Additional formative research in August to September 2015 aimed to evaluate self-testing accuracy among a convenience sample of female sex workers attending a Sisters clinic in Harare for HIV testing. Women were provided with validated pictorial instructions for use and a video demonstration. Self-test results were compared with confirmatory test results conducted by a health-care provider. 

In Malawi, little existing information was available on the population and lifestyle of female sex workers. Our formative research therefore aimed to explore the context and needs of female sex workers and to define the distribution approach for HIV self-testing. We conducted a rapid ethnographic assessment of female sex workers from January to June 2016. This assessment was followed by a participatory workshop in September 2016 with various stakeholders, including female sex workers, to present the results of formative research and invite input on the proposed peer-distribution model.

### Implementation phase

As a first step towards implementation of routine HIV self-testing in Zimbabwe, we conducted an observational study from November 2015 to April 2016 of all female sex workers who presented at the Sisters clinic in Harare. We assessed their preferences for HIV self-testing versus standard provider-delivered testing. We also carried out in-depth interviews with women selected from among those who opted for self-testing, to explore their preferences around distribution and support strategies.

In Malawi the peer-distribution model proposed in the formative research was implemented, whereby self-testing kits are distributed by other sex workers. During the implementation phase, from February 2017 to July 2017, we conducted a descriptive analysis of the uptake of self-testing kits and use of post-test support. We explored the acceptability and feasibility of the peer-distribution model though a process evaluation with focus group discussions with female sex workers and peer distributors. Once self-testing distribution was initiated, we established a parallel system for social harm monitoring and reporting through cross-sectional surveys and event diary reporting by female sex workers who received test kits, carried out from February 2017 to November 2017. 

### Scale-up phase

To date, only Zimbabwe has scaled-up HIV self-testing services, since October 2016. Distribution via community outreach began in September 2017 and secondary distribution of self-test kits for sexual partners was introduced in October 2017. We analysed routine programme data through September 2018 to describe uptake of self-testing by female sex workers and the number of self-test kits which female sex workers accepted for secondary distribution to their regular partners or clients. During this period, we explored additional approaches to HIV self-testing service provision in Zimbabwe through focus group discussions with stakeholders, including female sex workers, peer educators and service providers.

## Results

### Formative assessment phase

#### Zimbabwe

In the initial formative assessment, we found high acceptability of self-testing among female sex workers. Of 2722 women interviewed, 2069 (76%) reported being somewhat or very interested in testing themselves (versus not at all interested) and 1960 (72%) thought that if self-testing were available they would test more often. 

We found high accuracy of HIV self-testing when combined with pictorial instructions for use and an instructional video. Nine of the 40 female sex workers who participated in the accuracy study had reactive self-tests and all of these were confirmed HIV-positive (sensitivity: 100%; 95% confidence interval, CI: 88–100%). Thirty-one women tested HIV-negative with both HIV self-testing and confirmatory testing (specificity: 100%; 95% CI: 83–100%). When we consider one report of an unsure self-test result as false positive, specificity is 96.8% (95% CI: 66–100%).

#### Malawi

During the rapid assessment in Malawi we identified three categories of female sex worker: bar-based, street-based and home-based. Street-based female sex workers were hard to reach for services, although, along with home-based workers, were more independent and tended to have stronger support networks than bar-based workers. All female sex workers were highly mobile, following periodic transaction sex markets, although bar-based female sex workers were less so. Alcohol use was common and was a feature of transactions and negotiations with clients.

Women’s preference was for distribution of HIV self-testing kits through peers rather than general population clinics. However, some women expressed reservations that other sex workers’ knowledge of the use of HIV self-testing could be used against them when competing for clients. Concerns about how to securely store kits led to the decision for weekly stocks to be delivered to peer distributors, although this proved logistically difficult to maintain. There were also concerns about whether peer distributors would maintain confidentiality.

### Implementation phase

#### Zimbabwe

In the 6-month observational study of implementation of HIV self-testing in Zimbabwe, 325 (54%) of the 607 female sex workers who participated opted for HIV self-testing over provider-delivered testing (Table 2). Almost all women (313; 96%) opted to self-test onsite, rather than take the test away. In structured interviews with women who self-tested, acceptability of self-testing was high, with all women reporting the test was not difficult to use and 320 (98%) comfortable learning their result without a provider being present (Table 3). All the women said they would recommend HIV self-testing to family and friends, and 262 (81%) would recommend to clients. Of the 98 (30%) women with reactive HIV self-testing results, 97 (99%) had attended post-test services within 2–4 weeks (the time of the follow-up questionnaire). Among women testing negative, all wanted HIV self-testing to be available to them in the future, with 222 (98%) preferring to re-test at home, with or without someone else present. In open-ended questioning, where more than one response could be provided, women thought HIV self-testing kits should be distributed via a clinic (202 women; 62%), pharmacy (59; 18%), peers (46; 14%) or workplace (42; 13%). Suggestions for promotion of HIV self-testing were via mass media, pamphlets or posters at beer-halls and clinics, and peer education and outreach. Some women (124; 38%) thought that coercing individuals into testing was possible if HIV self-testing became widely available.

**Table 2:** Baseline characteristics among female sex workers opting for HIV self-testing in the implementation phase in Zimbabwe and Malawi

Characteristic	Value	
	Malawi (*n* = 268)	Zimbabwe (*n* = 325)
**Age, mean years (range)**	25 (NA)	29 (18–62)
**Marital status, no. (%) of women**		
Married or living as married	78 (29.1)	6 (1.9)
Never married	98 (36.6)	34 (10.5)
Separated or widowed	92 (34.3)	40 (12.3)
Divorced		245 (75.4)
**Education, no. (%) of women**		
No formal education	45 (16.8)	26 (2.6)
Primary school	128 (47.8)	217 (21.7)
Secondary school	91 (34.0)	692 (69.2)
Tertiary education	4 (1.5)	65 (6.5)
**Food insecure in past month, no. (%) of women**		
Never	NA	129 (39.7)
Sometimes	NA	115 (35.4)
Often or always	NA	81 (24.9)
**Ever tested for HIV, no. (%) of women**	232 (86.6)	305 (93.9)
**Clients in last week, median no. (range)**	NA	4 (0–60)
**How much of income is through sex work, no. (%) of women**		
Less than half	NA	131 (40.3)
More than half but not all	NA	42 (12.9)
All	NA	152 (46.8)
**Used condom at last sex, no. (%) of women**	NA	233 (71.7)

HIV: human immunodeficiency virus; NA: not applicable.

Notes: We analysed questionnaire data from the observational study in Zimbabwe (November 2015 to April 2016) and selected comparison data from a nested sub-study of HIV self-testing peer-led distribution in Malawi (February 2017 to July 2017). The questionnaires for the two research studies were developed independently and therefore do not align perfectly. We present data from Zimbabwe and where possible include comparable data from Malawi when a similar question was asked.

**Table 3:** Acceptability of self-testing among female sex workers opting for HIV self-testing in the implementation phase in Malawi and Zimbabwe

Characteristic	No. (%) of women	
	Malawi (*n* = 186)	Zimbabwe (*n* = 325)
**Tested at the clinic site**	NA	313 (96.3)
**Tested with someone else present**	132 (70.1)	4 (1.2)
**Reported self-test not at all hard to use**	NA	325 (100.0)
**Test result**		
Positive	61 (32.8)	98 (30.2)
Negative	116 (64.0)	226 (69.5)
Don't know/invalid	6 (3.2)	1 (0.3)
**Trusted that self-test result was correct**	NA	325 (100.0)
**Comfortable learning test result without a provider present**	NA	320 (98.5)
**Among those who tested HIV-positive, sought post-test services**	43 (70.5)	97 (99.0)
**Among those who did not test HIV-positive, would self-test again**	NA	227 (100.0)
**Among those who did not test HIV-positive, would want next test to be self-test**	96 (51.6)	226 (99.6)
**If self-tested again, would like to test**		
In own home	NA	317 (97.5)
In home of friend or partner or relative	NA	1 (0.3)
In a clinic or hospital	NA	7 (2.2)
**If self-tested again, would like to test**		
Alone	NA	166 (51.1)
With someone else present	NA	159 (48.9)
**Would recommend self-testing to friend or family**	178 (95.7)	325 (100.0)
**Would recommend self-testing to a client**	NA	262 (80.6)
**Would like self-testing to be regularly available**	NA	325 (100)
**How hard was it to understand what services to attend after self-testing?**		
Not at all hard	NA	325 (100)
Somewhat hard	NA	0
Very hard	NA	0

HIV: human immunodeficiency virus; NA: not applicable.

Notes: We analysed data from the observational study in Zimbabwe (November 2015 to April 2016) and selected comparison data from a nested sub-study of HIV self-testing peer-led distribution in Malawi (February 2017 to July 2017). The questionnaires for the two research studies were developed independently and therefore do not align perfectly. We present data from Zimbabwe and where possible include comparable data from Malawi when a similar question was asked.

In qualitative analysis from in-depth interviews with 31 female sex workers, women reported recognizing their high risk of being HIV-positive and wanting to access onsite confirmatory testing and ART if needed. The women were also concerned that others would find out if they took the self-test kit home. Female sex workers reported being attracted to HIV self-testing for its novelty and the chance to be among the first to try it. They endorsed the video instructions in the local language as easy to understand. Although most women would recommend self-testing to clients, the nature of the relationship was critical in this respect, with fears of discouraging irregular clients and otherwise little motivation to discuss HIV self-testing during a brief sexual encounter. A minority of participants indicated they would find client self-testing informative as a measure of transmission risk, which would help maintain their HIV-negative status. Willingness to discuss HIV self-testing with regular sexual partners and clients was higher.

#### Malawi

Implementation in Malawi started with recruitment and training of 28 peer distributors in January 2017 including one lead peer-distributor from each district. Eight peer educators were dropped due to poor performance, and five new distributors were trained in their place.

Over the 6-month study period, a total of 5281 test kits were distributed by 25 peer distributors. On average, each peer distributed 211 test kits, mostly to young female sex workers aged 16–24 years (3010 kits; 57%); 264 (5%) kits went to female sex workers older than 35 years. Kits were distributed to venue-based (3644 kits; 69%), home-based (1003; 19%) and street-based (634; 12%) female sex workers. Nearly one-third (1592; 30%) of tests were done under observation of a peer distributor, 2240 (42%) were administered by peer distributors and 1449 (27%) were self-administered in private. Heavy reliance on peer distributors could be explained by the low literacy in this group of female sex workers (Table 2).

Almost all (4964; 94%) tests were used within 7 days. Based on late reading of 4096 (78%) returned test kits, an estimated 1352 (33%) were reactive (Table 3). However, this is likely imprecise because late reading has been associated with inflated estimates of positivity.^17^ Linkage to support services could only be verified for 61 female sex workers, likely due to a combination of reporting issues. Problems included female sex workers presenting at a range of health-care facilities, making tracking difficult and high rates of previously known HIV-positive status through the standard HIV testing services provided by Pakachere Institute.

In the cohort study of social harm, none of the 265 female sex workers who self-tested reported any experiences of social harm, despite there being systems in place to do so. However, the women’s diary reports noted 29 events of coercive testing and 28 forced disclosures between female sex workers and established intimate partners, family members, peers or peer distributors. Some female sex workers reported regretting testing. Some women experienced physical violence, mostly perpetrated by an established partner, linked to disclosure of results or requesting that a partner also test and typically in the context of pre-existing relationship violence. No women reported breaches in confidentiality by peer distributors. Peer distributors reported experiences of social stigma, questioning of their credentials and low-grade violence by female sex workers who self-tested and other community members.

Analysis of focus group discussion data from the process evaluation indicated that many peer distributors assumed anyone refusing HIV self-testing was likely already known to be HIV-positive. Therefore, we anticipate that re-testing among known HIV-positive women already linked to care was common, as has been identified in previous research in Malawi.^18^ Tracking linkage to post-test services was also likely underreported as female sex workers used aliases at ART services, frequently attended services other than those provide through Pakachere Institute and were highly mobile. Peer distributors managed the demonstration of HIV self-testing with ease, but high demand was hindered by difficulty in resupplying test kits weekly. We identified additional distribution challenges including alcohol-related issues for some female sex workers, and concerns that the peer distributors wanted to steal their clients.

The female sex workers expressed excitement that HIV self-testing kits were distributed through peers (Box 2). They considered this approach better than clinic-based testing, describing it as convenient, confidential, flexible, less costly, easy to use and less intrusive for individuals in high-risk occupations. The women perceived peer distributors as individuals who maintained the voluntary nature of the process and who understood and safeguarded the privacy of self-testers, and this made peer distributors feel accepted and respected. However, coverage of a peer-led model was perceived to be restricted to people who encountered the distributor, therefore ignoring hidden female sex workers and those working in high-risk environments. Distribution of test kits was not possible where potential testers were drunk or were working.

Box 2 **Perspectives on HIV self-testing for female sex workers in Malawi and Zimbabwe**

We present some perspectives from qualitative research of female sex workers and other stakeholders on acceptability of HIV self-testing, linkage to post-test services and HIV self-testing distribution models.

**Formative assessment phase**

Malawi

• Female sex workers:

“HIV self-testing saves transport because you can do it at home, as opposed to the blood based which requires one to go to the hospital.”

“You don’t wait for anyone to tell you your status. You know your own status therefore it’s more private and it’s up to you whether you tell someone or not.”

Zimbabwe

• Female sex workers enrolled in Sisters programmes:

“If they [female sex workers] have been taught about self-testing, what it is, how it is done, it [testing uptake] would increase because most people like their privacy a lot.” 20-year-old sex worker of 2 years

“I think that if a person wants to self-test, they should come to the clinic and be given a kit here, right here.” 37-year-old sex worker of 8 years

**Implementation phase**

Malawi

• Female sex workers:

“This kit is comfortable and easy to use. The self-testing process is better because it doesn’t involve any piercings hence, does not hurt.”

“They [peer-distributors] were gentle with us and told us that they are not forcing anyone to test. It was our decision.”

“They [peer-distributors] encouraged those of us that were afraid to test to finally get an HIV test.”

“They [peer-distributors] didn’t ask us our results when giving them the envelopes. It was up to us to disclose. They should be maintained because they kept our information safe.”

**Scale-up phase**

Zimbabwe

• Female sex workers not enrolled in Sisters programming:

“You can just stay at home hesitating, to get medication because there is no one encouraging you.” 25-year-old sex worker

“I want to be caught [test positive] and then I can start taking the pills before I am bed ridden if I have it.” 28-year-old sex worker

“So if you are testing yourself, and you see your results, if you test positive you then access treatment, alone, you do it yourself, just doing it for yourself, without anyone knowing that, you are positive.” 28-year-old sex worker

• Female sex workers enrolled in Sisters programming:

“As soon as you leave, she [the peer-educator] will talk about you saying, ‘You see the lady who just left? I have given her a self-test kit’.” 40-year-old sex worker of 10 years

“Hairdressers should not even [distribute]: they will obviously remain behind and talk [about us].” 20-year-old sex worker of 3 years

“We don’t want to get from hairdressers, their husbands are our clients.” 29-year-old sex worker of 8 years

“If it’s me, I will give [vouchers] to my workmates at our workplace, as sex workers.” 22-year-old sex worker of 2 years

• Peer educators:

“As peer educators, our role first of all is to provide counselling, educating the person we would want to give self-testing kits to.” 26-year-old sex worker of 3 years

“I view it [self-testing] as a good thing, even our clients who are reluctant to visit health centres… they might accept this one and see their results on their own.” 36-year-old sex worker of 8 years

“A hairdresser is a person just like me. Maybe I would have snatched her partner or client the previous day… She might not give me the kit even if I want it because… she will be frustrated with me. Plus, they can take these kits and misuse them giving to her relatives instead of giving to sex workers.” 37-year-old sex worker of 8 years

• Hairdressers involved in condom promotion:

“For me to be motivated to do the work, if you give me money…not to give me a salary as someone who is employed, just some money for me to value your work, it gives me strength for me to do it wholeheartedly.” 30-year-old hairdresser of 15 years

HIV: human immunodeficiency virus.

### Scale-up phase

#### Zimbabwe

In the scale-up phase in Zimbabwe, data on distribution of test kits from seven Sisters clinics and via community outreach showed that over the distribution period a total of 19 251 women presented for testing and 12 071 (63%) opted to self-test. Among self-testers, 11 347 (94%) tested onsite. Among those testing onsite, 1616 (14%) went for confirmatory testing, of whom 1443 (89%) were confirmed positive. Of the 7180 women who opted for provider-delivered testing, 1482 (21%) were confirmed positive. All those confirmed positive were referred for HIV care. Over a 12-month period, 12 061 female sex workers were offered test kits for their regular partners or clients. A total of 972 (8%) women accepted a total of 1056 test kits, with 896 (92%) accepting one kit, 68 (7%) accepting two kits and 8 (1%) accepting the maximum three kits.

Additional approaches to provision of HIV self-testing were explored in 15 focus group discussions with 7–10 participants each. Willingness to distribute test kits was high among peer educators, care promoters and hairdressers, with some offering to also provide pre-test counselling or door-to-door distribution. Most female sex workers attending Harare Sisters services were against the distribution of kits by peer educators or alternative providers. Similar to Malawi, they expressed concerns that peers would not maintain confidentiality. They preferred distribution by Sisters staff, with onsite self-testing available (Box 2).

We also conducted four focus group discussions among 39 female sex workers who had never attended Sisters services. These women had limited awareness of HIV self-testing, but thought it would increase the uptake of testing. Views on distribution of test kits varied, but peer educators (with whom this population had limited contact) were considered possible distributors. In keeping with their limited engagement with clinic services, these women expressed privacy concerns and greater distrust of these services.

## Discussion

This comparison of implementing HIV self-testing for female sex workers in two high-burden countries highlights differences in approach, as well as similarities, both of which reflect the underlying service context. In Zimbabwe there was a detailed understanding of the context of female sex workers and dedicated services reaching a high number of women since 2009. This setting offered a ready framework to implement and evaluate HIV self-testing strategies. Women’s preferences for how to access HIV self-testing differed substantially according to how supportive the existing programme infrastructure was. When offered through a strong female sex worker programme, clinic-based HIV self-testing distribution with onsite self-testing was preferred by the women. In contrast, peer-distribution models were favoured by female sex workers in Malawi and female sex workers in Zimbabwe who were not engaged in the programme. The advantages of clinic-based distribution were that uptake of onsite self-testing was high and almost all women reported linking into post-test services. An advantage of peer distribution was the potential to reach female sex workers not previously linked into clinical services.

Malawi, in contrast to Zimbabwe, had little prior experience with programming for female sex workers, and a rapid ethnographic assessment resulted in a better understanding of the context and needs of female sex workers. This assessment provided valuable insight into the optimal distribution strategy for self-testing kits, given the more limited female sex-worker-friendly services. Malawi used a peer-led approach, which despite some reservations, was considered feasible and acceptable to female sex workers. While HIV self-testing proved acceptable in general and specifically this distribution model, a rigorous evaluation of social harms indicated that some women felt coerced into testing or forced to tell their partners.^19^ Understanding how these events occurred and ways to prevent adverse outcomes is critical in HIV self-testing, as with any HIV testing services and particularly for female sex workers who often face high levels of stigma, discrimination, exclusion and violence. Supply-side logistics were challenging, leading to lower-than-anticipated distribution of self-testing kits and poor ability to demonstrate linkage to post-test care. Similar to HIV self-testing programmes elsewhere, implementers had concerns about theft or inappropriate storage of HIV self-testing kits, resulting in supply shortages. Supporting the peer-distributor model, a cluster-randomized trial in Uganda comparing distribution strategies showed that a peer-distribution model reached more individuals than did HIV self-testing provided via vouchers for redemption at non-specialist clinics.^9^

Important to our approach in both countries was the involvement of female sex workers at each step in the process, to guide programme development and scale-up. Health ministries and other stakeholders were also closely involved. This involvement resulted in different implementation strategies and highlighted the importance of not transferring implementation models from one setting to another without formative work. Zimbabwe’s clinic-based strategy was readily scaled-up nationally. As a single distribution strategy is unlikely to reach everyone, additional strategies to reach a broader segment of the population with HIV self-testing services, including providing kits for partners, were then implemented. Qualitative research conducted throughout planning and implementation in both countries enhanced understanding of the needs, opinions, and experiences of female sex workers regarding HIV self-testing programmes.

In Zimbabwe, we demonstrated high accuracy with unassisted HIV self-testing when combined with instructions for use and an instructional video.^15^ General literacy and HIV knowledge is generally high among female sex workers in Zimbabwe, in contrast to Malawi. Other studies have shown differing results. In Uganda, even with an in-person demonstration, sensitivity and specificity of HIV self-testing were below 70%, primarily due to difficulties interpreting the results.^20^ Accurate testing in one context cannot therefore be generalized to female sex workers in all settings. Ensuring instructions for use provided with test kits can support accuracy of testing is an important pre-implementation step.

While Malawi has not explored secondary distribution of HIV self-testing through female sex workers, Zimbabwe demonstrated success with this strategy. Data from Kenya also support this strategy,^12^ which can infiltrate high-risk social and sexual networks. As with primary distribution, kits can be disseminated through clinics, peers, community outreach and voucher systems. Important considerations are acceptability, accuracy, safety and subsequent uptake of HIV care, and prevention services. The risk of social harm with secondary distribution strategies may be greater and strategies to mitigate this are required.

Measuring and evaluating linkage to post-test services is a common challenge with HIV self-testing and among female sex workers this is no different. One of the strongest advantages of facility-based distribution is the ease and completeness of monitoring confirmatory testing. In contrast, data for women and their partners using HIV self-testing kits offsite was incomplete, and less reliable. However, a key tenet of self-testing is the privacy it affords, which was highly valued in these criminalized and vulnerable populations in both countries. Access to private HIV self-testing should not be undermined by over-eager efforts to collect and track results, linkage data or identifying information. Additionally, peer distribution of HIV self-testing kits is not a substitute for providing comprehensive services for female sex workers, which appear to be strongly preferred when available. Peer and secondary distribution strategies may then act to stimulate demand for comprehensive HIV services.

Our implementation data has some limitations. Methods across countries were not identical, resulting in challenges for comparing and generalizing the results. We cannot be certain of the representativeness of our participants. Zimbabwe female sex workers were, by definition, more exposed to services than those in Malawi. Our data do not adequately capture linkage into post-test support services, a common limitation in HIV self-testing programmes. Importantly, our research looked only at female sex workers and did not explore the unique needs of other sex worker populations.

Bringing HIV self-testing to scale for female sex workers has the potential to improve awareness of HIV status, the first step for prevention and care services. Ensuring female sex workers are adequately engaged in HIV prevention and care is important both for their own health and well-being, and for the broader public health.^21^ Investment in these services is likely to be crucial to controlling the HIV epidemic. Our studies highlight important gaps and questions that remain to be answered (Box 3). As for other interventions for female sex workers, an enabling environment is essential, including decriminalization of sex workers and programmes that build community empowerment.^22^ HIV self-testing has the potential to be one of the tools to facilitate this process.

Box 3 **Lessons learnt and research gaps identified in implementation and scale-up of HIV self-testing for female sex workers in Malawi and Zimbabwe**

**Lessons learnt**

- Female sex workers must be involved at all stages of planning and implementation.
- Female sex workers can test themselves accurately if adequately supported, but this may depend on the context or on women’s levels of education and literacy.
- Acceptability of HIV self-testing by female sex workers is high, mainly because it overcomes some of the barriers they face, or perceive they face, when testing through general population or targeted services.
- The desire of programme implementers to measure linkage to prevention and care needs to be balanced against female sex workers’ desire for privacy and confidentiality, which is key to the high acceptability of HIV self-testing in this population.
- Female sex workers have the potential to reach other hidden, high-risk populations using secondary distribution.
- Despite little evidence of social harms, distribution models need to consider concerns that coercion to test might occur. Systems for monitoring and mitigating social harm need to be in place.
- Models of distribution will likely need to be adapted to each setting and with explicit input from sex workers about what is likely to work in their context.

**Research gaps**

- How should we optimize measurement of linkage to prevention and care without compromising the key feature that makes HIV self-testing acceptable i.e. desire for privacy and confidentiality?
- Can distribution of HIV self-test kits be done in a way that builds community trust and social cohesion, and thereby improves female sex workers’ engagement with HIV and other health-care services?
- How should we adapt distribution and support to increase awareness of HIV status, and linkage to prevention and care among younger women who sell sex?
- What are the optimal systems for monitoring and mitigating social harms?
- How could newer and improved HIV self-testing kits be used to support implementation of pre-exposure prophylaxis among female sex workers?
- How do we optimize secondary distribution to reach hidden high-risk populations in a way that is safe, maximizes impact and is cost–effective?
- What is the relative cost–effectiveness of different approaches to distribution of HIV self-test kits?
- How do we optimize HIV self-testing programmes to address the specific needs of other sex-worker groups, including male and transgender sex workers?

HIV: human immunodeficiency virus.
